# Community Outreach Panel Explores and Addresses Higher Rates of COVID-19–Related Deaths in the African American Population

**DOI:** 10.31486/toj.20.0063

**Published:** 2020

**Authors:** Yvens Laborde, Olivia Manayan

**Affiliations:** ^1^ Medical Director, Global Health Education, Medical Director, Public Health, Ochsner Clinic Foundation, New Orleans, LA; ^2^ The University of Queensland Faculty of Medicine, Ochsner Clinical School, New Orleans, LA; ^3^ Research Fellow, Ochsner Clinic Foundation, New Orleans, LA

## TO THE EDITOR

In Louisiana, African Americans are disproportionately affected by poor health outcomes compared to whites with similar health conditions.^1^ The novel coronavirus (COVID-19) has proven to be no exception, with the African American population accounting for 70.5% of COVID-19–related deaths, despite only comprising 32.2% of the state's population.^2^ This disparity is thought to be multifactorial, with many of the factors that contribute to health inequity in the African American population also contributing to the morbidity and mortality rates seen in this same population with the novel coronavirus. These factors, which include higher rates of poverty and housing density; lower rates of stable, salaried jobs that permit work-from-home arrangements; and the burden of preexisting, chronic medical conditions, effectively equate to an inability for many members of this community to practice social distancing.

To gain further insight into how healthcare professionals can address these factors, Drs Yvens Laborde and Olivia Manayan, in collaboration with the Regular Baptist Church of New Orleans, organized a question and answer panel (Figure) with the aims of (1) providing accurate, up-to-date, evidence-based information about COVID-19 to the public in a way that was approachable and accessible, (2) answering questions posed by members of the community, and (3) gaining a better understanding of the root causes of inequities in the healthcare system. The panel was administered through the Zoom meeting platform and lasted approximately 60 minutes, with 36 members of the community participating.

**Figure:**
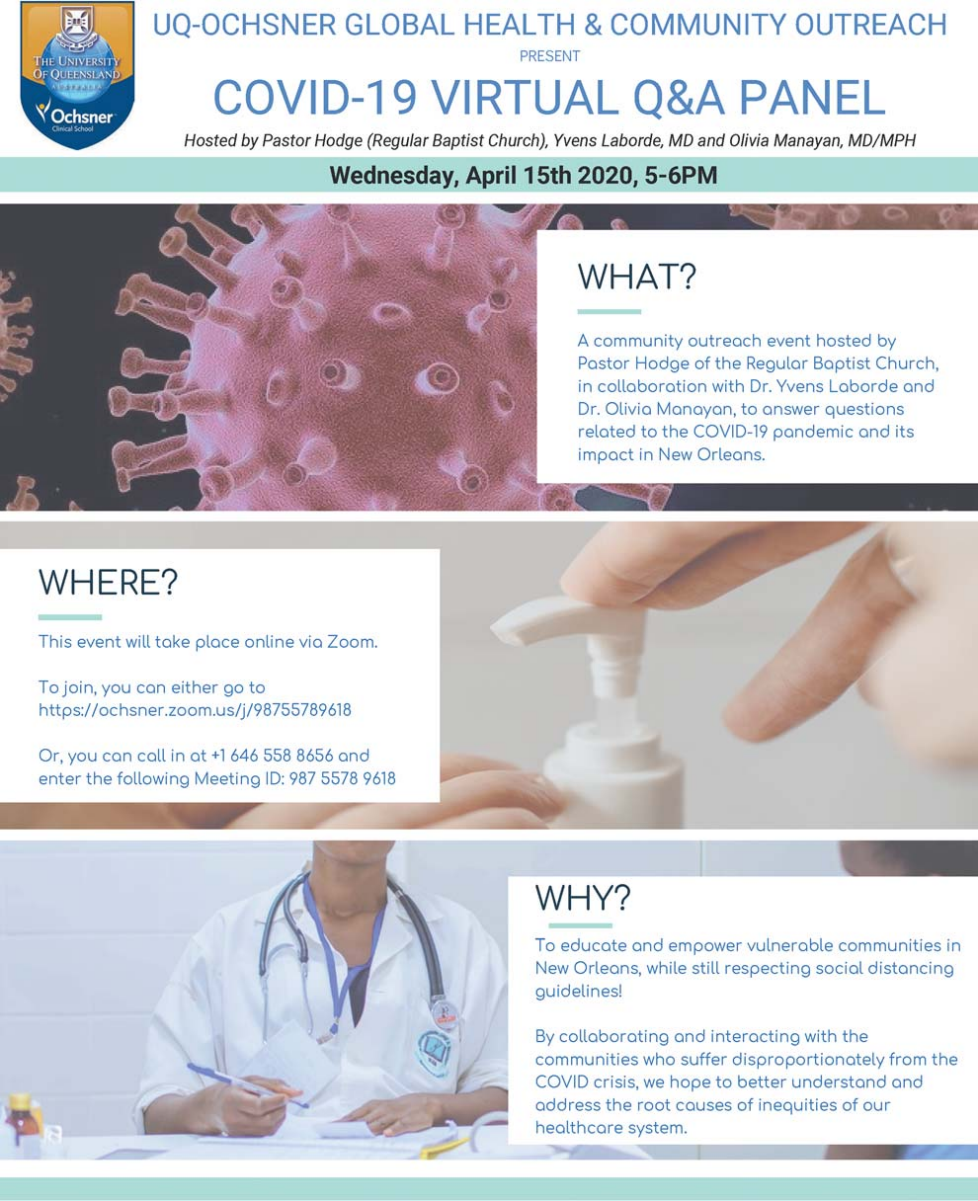


Among the themes discussed were safe social distancing practices, current guidelines for treatment and testing of COVID-19, tackling social isolation in the home and intensive care unit setting, and guidelines for quarantining at home. Participants expressed interest in participating in clinical trials for novel treatments for the coronavirus and for at-home monitoring of symptoms such as blood pressure and blood oxygen saturation, particularly those who had preexisting comorbidities such as hypertension, diabetes, obesity, and chronic obstructive pulmonary disease. Participants sought clarification about articles they had encountered in the media and asked about the use of extracorporeal membrane oxygenation and hyperbaric oxygen chambers as treatments and the use of humidifiers combined with hydrogen peroxide as air sanitation devices.

Participants expressed the desire for easier and greater access to community testing. One important insight was that not all individuals have access to a vehicle; therefore, the participants expressed a strong desire for the availability of walk-up testing. Another challenge is multigenerational households and the need for social support to obtain alternate temporary housing options for COVID-19–positive patients being cared for at home. The participants also discussed the heavy financial and mental burdens that the stay-at-home orders were having on them individually and on their communities.

Gaining first-hand perspectives from individuals at higher risk of mortality from the novel coronavirus is necessary to understand the inequities in healthcare and to form effective strategies to combat these inequities. Additional community engagement and outreach activities are needed to further our understanding and to improve the accessibility of healthcare and information for high-risk communities. We hope to engage in more community-based interventions that incorporate the feedback provided during the first session, such as increased at-home monitoring using smart devices that digitally share measurements (eg, oxygen saturation monitors and thermometers). Further, all such community-based outreach activities should take into account the importance of meeting context and setting, particularly focusing on the increased effectiveness of integrating outreach into preexisting community groups such as churches or vocational groups. Innovative methods of community outreach that are culturally sensitive can provide a powerful platform to engage, empower, and improve health outcomes for African Americans and other at-risk communities.

As Martin Luther King famously stated, “Of all the forms of inequality, injustice in health is the most shocking and the most inhuman because it often results in physical death.”
